# ‘I had a bigger cancer risk than I thought…’: The experience of receiving personalized risk information as part of a skin cancer prevention intervention in the college setting

**DOI:** 10.1111/hex.13601

**Published:** 2022-10-12

**Authors:** Hannah L. Brady, Jada G. Hamilton, Kimberly A. Kaphingst, Jakob D. Jensen, Wendy Kohlmann, Bridget G. Parsons, Helen M. Lillie, Ali P. Wankier, Heather J. Smith, Douglas Grossman, Jennifer L. Hay, Yelena P. Wu

**Affiliations:** ^1^ Cancer Control and Population Sciences Division, Huntsman Cancer Institute University of Utah Salt Lake City Utah USA; ^2^ Department of Psychiatry & Behavioral Sciences Memorial Sloan Kettering Cancer Center New York New York USA; ^3^ Department of Medicine Memorial Sloan Kettering Cancer Center New York New York USA; ^4^ Department of Communication University of Utah Salt Lake City Utah USA; ^5^ Department of Dermatology University of Utah Health Sciences Center Salt Lake City Utah USA

**Keywords:** health behaviour, intention, motivation, risk communication, skin cancer

## Abstract

**Background:**

Diagnoses of both melanoma and nonmelanoma skin cancers are becoming increasingly common among young adults. Interventions in this population are a priority because they do not consistently follow skin cancer prevention recommendations.

**Objectives:**

The goal of the current study was to examine college students' perspectives on and experience with receiving a skin cancer prevention intervention that provided personalized skin cancer risk feedback in the form of an ultraviolet (UV) photograph, the results of genetic testing for common skin cancer risk variants, and/or general skin cancer prevention education.

**Methods:**

Qualitative interviews were conducted with 38 college students who received a skin cancer prevention intervention. The interview covered students' feelings about their personal skin cancer risk information, the impact of the intervention on their skin cancer risk perceptions, actions or intentions to act with regard to their sun protection practices and feedback for improvement of the intervention content or delivery.

**Results:**

Participants reported that different intervention components contributed to increased awareness of their sun protection behaviours, shifts in cognitions about and motivation to implement sun protection strategies and reported changes to their skin cancer prevention strategies.

**Conclusion:**

Our findings indicate that college students are interested in and responsive to these types of multicomponent skin cancer preventive interventions. Further, students demonstrate some motivation and intentionality toward changing their skin cancer risk behaviour in the short term.

**Patient or Public Contribution:**

Participants involved in this study were members of the public (undergraduate students) who were involved in a skin cancer prevention intervention, then participated in semistructured interviews, which provided the data analysed for this study.

## 1 INTRODUCTION

Skin cancer is the most commonly diagnosed cancer in the United States and the incidence of skin cancer, including melanoma, the most deadly form of skin cancer, is increasing worldwide.^1^, ^2^, ^3^, ^4^, ^5^ Melanoma is the third most common cancer among young adults and diagnoses of nonmelanoma skin cancers are increasing, especially in women.^6^ Ultraviolet (UV) radiation from sun exposure and artificial sources such as indoor tanning beds is a key risk factor for skin cancers.^7^, ^8^, ^9^, ^10^, ^11^, ^12^ Current skin cancer prevention recommendations include sun protective behaviours such as wearing protective clothing, applying sunscreen to exposed skin, seeking shade when outdoors, avoiding outdoor exposure during peak hours (10 am to 4 pm), and avoiding tanning.^13^, ^14^

College‐age students are an important target population for skin cancer prevention interventions because, despite the downward trend in indoor tanning rates, they still report frequent sunburns or unintentional suntans due to inconsistent use of recommended sun protection methods and high levels of skin cancer risk behaviours.^15^, ^16^, ^17^, ^18^ Given the critical life developments that occur between the ages of 18 and 25,^19^, ^20^ targeting skin cancer prevention interventions in this setting should be a high priority.

Prior studies in skin cancer prevention have shown that communication strategies that focus on appearance, such as providing information about the damaging effects of UV radiation exposure (e.g., ageing, sunspots, wrinkles, etc.) and the use of stock or personalized UV photographs that show underlying skin damage, contribute to increased use of sun protection strategies and a reduction in tanning behaviours among college students.^21^, ^22^, ^23^, ^24^, ^25^, ^26^, ^27^, ^28^, ^29^, ^30^, ^31^, ^32^, ^33^ Studies have also examined whether interventions that highlight individual information about skin cancer risks, such as a UV photograph or personal risk assessment effectively improve sun protection or tanning behaviours in college students. In these studies, researchers found some improvement in sun protection behaviours in the intervention group.^25^, ^26^, ^27^ While these prior studies examined quantitative outcomes and the effects of the intervention on subsequent behaviours,^30^, ^31^, ^32^, ^33^ few have examined students' overall reactions to intervention content and delivery. Studies that did explore the intervention experience from participants' perspectives were conducted among children and adolescents in school or community settings or with their parents.^26^, ^34^, ^35^, ^36^, ^37^, ^38^ Little is known about college‐aged participants' perspectives on receiving skin cancer prevention interventions, which could provide context for understanding intervention engagement and effects, inform future intervention development and targeting for this population and aid in the examination of intervention mechanisms. These perspectives warrant examination in a qualitative study.

We recently piloted a novel multicomponent skin cancer preventive intervention that provided personalized skin cancer risk information to undergraduate students in the form of a personalized UV photograph and the results of genetic testing for germline variations in the melanocortin‐1 receptor (*MC1R*) gene.^39^, ^40^ The results of the latter indicated whether an individual was at average risk (i.e., 1 in 100) or higher‐than‐average risk (i.e., 3 in 100) for the development of melanoma.^41^, ^42^, ^43^ Existing skin cancer prevention studies that explored the addition of genomic risk information as an intervention component yielded results that were mixed across study samples; however, the overall results are promising in the general population. For example, Lacson et al.,^44^ and Smit et al.,^45^ found that *MC1R* genetic testing and precision prevention materials increase the practice of sun‐protective and early detection behaviours. Hay et al.,^46^ found that *MC1R* testing and precision prevention materials increased sun protection among those who were less aware of their skin cancer risk. For our pilot study, we found that the interventions were associated with improvement in sun protection and tanning behaviours, with no one combination of components significantly better than the others in improving sun protection and tanning behaviours.^39^ Given the mixed outcomes of studies on current risk communication methods among this population,^22^, ^23^, ^24^, ^25^, ^26^, ^27^ it is possible that college students require more novel forms of risk communication to motivate behaviour change.^47^

The goal of the present study, a qualitative analysis of the results from the aforementioned pilot study, is to gain insight into students' experience with a personalized skin cancer risk intervention. We sought to better understand how this intervention may have affected students' cognitions around current sun protection, tanning behaviours and individual risk perception and how these could motivate protective behaviour change among this population. The current study examined students' perspectives on their (1) experience with the specific intervention components; (2) internal reactions (emotional reactions to personal risk information, effect on individual risk perception, impact on motivation/intentions around sun protection) and external responses (communication with others, actions taken) to interventions received; and (3) recommended changes to intervention content and delivery.

## 2 METHODS

### 2.1 Participants

In total, 38 college students completed open‐ended interviews (Table 1). Students were recruited from an undergraduate Communication course at a 4‐year public university and through advertisements on campus (i.e., paper flyers, ads on shuttle buses). Recruitment occurred in the fall and spring semesters. Students were eligible for inclusion if they were at least 18, were enrolled as a student at the university, had at least one sunburn in the last year, and/or reported intentional tanning at least once in the last year, and/or reported using sunscreen plus one other sun protection behaviour (protective clothing and shade) infrequently.^13^, ^48^ Students were excluded if they did not read or speak English or had a personal skin cancer history.

**Table 1:** Demographic characteristics of study participants (*N* = 38)

	*n* (%)^a^
Age (*M*, SD)	21.3 (1.9)
Sex	
Male	3 (7.9)
Female	35 (92.1)
Race	
White	34 (89.5)
Black or African American	1 (2.6)
Asian	2 (5.3)
Other/multiracial	1 (2.6)
Ethnicity	
Spanish, Hispanic or Latino/a	3 (7.9)
Marital status	
Married or marriage‐like relationship	6 (15.8)
Never married	32 (84.2)
Family income	
<$50,000	10 (26.3)
≥$50,000	18 (47.3)
Unsure	8 (21.1)
I would rather not report this	2 (5.3)
Fitzpatrick skin type	
I	3 (7.9)
II	12 (31.6)
III	14 (36.8)
IV	8 (21.1)
V	1 (2.6)
VI	0 (0)
Family history of melanoma	
Yes	14 (36.8)
No	10 (26.3)
Unsure	14 (36.8)
Personal history of cancer	
None	38 (100)

^a^ *n* and % reported for demographic variables unless otherwise noted.

### 2.2 Procedures

Students completed a baseline visit where they provided informed consent, completed assessments and were randomly assigned to one of four groups: education only (Group 1); education + UV photograph (Group 2); education + *MC1R* testing (Group 3) and education + UV photograph + *MC1R* testing (Group 4). During the baseline, the UV photographs were taken and students provided a saliva sample for *MC1R* testing depending on the group assignment. One month later, a second in‐person visit occurred where participants received the risk feedback intervention(s). The personalized UV photograph showed the student's face in visible light and UV light, which displayed underlying skin damage.^49^ *MC1R* testing feedback was provided as two icon arrays comparing twofold (3 in 100 vs. 1 in 100 risks) to average risk (carriers) or a single icon array showing average risk (noncarriers). All participants were given educational information on the importance of skin cancer prevention during their younger years as a means of decreasing skin cancer risk. Participants with complex questions about their results that extended beyond the scope of the study could be referred to a genetic counsellor from the local cancer centre for further clarification. One month after the second visit, participants were asked to complete an electronic survey. After the survey, participants were invited to participate in a 20–30‐min interview. Participants were offered course credit or a gift card.

The study enrolled 92 undergraduate students. Of those enrolled, 86 completed study visits and were invited to participate in the follow‐up interview, and 38 completed an interview (Group 1: *n* = 9; Group 2: *n* = 11; Group 3: *n* = 12; Group 4; *n* = 6). There were no significant baseline differences in demographic characteristics such as age, gender, race and family history of melanoma or in sun protection behaviours and sunburn occurrence between participants who completed an interview and those who did not. The interviews were audio‐recorded and transcribed for coding and analysis. All study procedures were approved by the University Institutional Review Board.

### 2.3 Measures and analysis

#### 2.3.1 Demographics

Participants were asked about their gender, race/ethnicity, marital status, income, family history of melanoma and personal history of cancer and skin type.^50^

#### 2.3.2 Participant interview

After the research assistant reviewed the participants' assigned intervention arm with the participant, the interview covered feelings about their personal skin cancer risk, the impact of the intervention on skin cancer risk perceptions, actions or intentions to act with regard to their sun protection behaviours, and suggestions for improvement of the intervention content and delivery (see Table 2).

**Table 2:** Qualitative interview guide

(1) In your own words, what information about your skin cancer risk did you receive from the RISE‐UP study?
Probe: Did you receive educational information?
Probe: Did you receive *MC1R* genetic test results?
If yes, probe: What did you learn about your skin cancer risk from those genetic test results?
Probe: Did you receive a UV photo?
If yes, probe: What did you learn about your skin cancer risk from your UV photo?
(2) How did you feel when you received the information about your skin cancer risk?
(3) Was this information about what you expected, or was it surprising?
Probe: What specifically met your expectations? What did you find surprising?
(4) To what extent did you find this information about your skin cancer risk to be new or novel?
Probe: What specifically about the information felt novel? What specifically felt familiar or like something you already knew?
(5) What did you do with the information about your skin cancer risk?
(6) Did you show or discuss the information with anyone? If so, whom?
(7) After you received the information about your skin cancer risk, how often did you think about it later?
Probe: When did you find yourself thinking about it? What, specifically, did you think about? How did you feel when you thought about this information?
(8) How satisfied or disappointed did you feel about the content of the information that you received?
Probe: Did you hope to receive something different?
Probe: What do you think could make the information better?

#### 2.3.3 Data analysis

The initial codebook was developed by a master's‐level research assistant (primary coder) and a bachelor's level research assistant (reliability coder) who utilized a thematic analysis approach to identify eight key categories (Table 3).^51^ An iterative procedure was used to finalize the codebook whereby independent coding of the same selection of six transcripts (this number was selected based on the number of transcripts required before new categories were no longer being identified) was completed by both coders. Coding discrepancies were discussed and resolved in collaboration with the study's principal investigators. The finalized codebook was used by the primary coder to code the remaining transcripts and to recode the initial six. Intercoder agreement for 20% of transcripts was 77%. The primary coder re‐examined all coded segments to identify subcategories.

**Table 3:** Summary of key categories

Key category	Subcategory	Exemplar quotations
Feelings/emotions	Negative emotional reaction	A little sad, a little disappointed in myself for not wearing sunscreen all the times I was supposed to. (Group 2)
	Positive emotional reaction	I was definitely relieved, but, again, I know it's not a guarantee, and so it doesn't mean that I'm fine to just do whatever and not wear sunscreen. I recognize that. (Group 3)
	Existing knowledge/expectation of skin cancer risk	Not that surprised, considering almost every—eight out of ten people in my family have had skin cancer. However, I didn't think that I would have as many sunspots underneath my skin as I did with the amount of sunscreen that I always put on my face. (Group 2)
	Affirmation/validation of current skin protective behaviours	I felt pretty good 'cause I was, like, ‘I do most of these things’. Some of them I don't do, but most of them I do, so I wasn't really worried about it, about getting sick, getting skin cancer. (Group 1)
	Urgency for action	I guess it was a little concerning, but not so much that I'd actually be anxious about it. More so that I was just kind of glad I was informed…. (Group 1)
	Increased confidence/self‐efficacy	I felt that this is a problem, but it can be fixed. That's how I felt that, yes, there's an issue here, but if we take proper precautions, like I learned, then it will be okay. (Group 2)
	No emotional reaction	I felt indifferent. I wasn't really worried. I think when I saw the picture of my skin, I still remained calm, and I didn't feel like there was much to be nervous about. (Group 4)
Information gained through study	Confirmation of existing knowledge	…I felt I already knew some of the more basic, rudimentary information, that you should wear sunscreen, and you should try to cover your arms and legs to protect them, but I didn't know to what extent or to what importance, and what effect it had on our skin…. (Group 4)
	Knowledge gained was opposite of existing knowledge	I thought I was higher at risk before the study because my father has had skin cancer, as well as one of my grandparents, and so I thought it was a genetic thing … From the genetic testing, I found out that I'm actually not at risk for it, at least not the genetic standpoint. (Group 3)
	New information gained from study	I received information regarding my appearance, I guess. I have blue eyes. I tan easily. I have freckles. I have fair skin. I didn't really know that all of those things combined increased the risk for skin cancer. That's what I learned, is that, based on my genetics like physical appearances, I would have more of a risk. (Group 1)
	Introduction to novel screening methods	The saliva test, that was all new info. I didn't know I could be tested for a genetic marker for skin cancer, so that was really new and cool. I like that opportunity. (Group 4)
Communication/information sharing	Specific conversations, proactive communication	I shared it with everybody I knew because I was so fascinated with especially the UV photograph that I just kind of wanted to … make them aware of what theirs could look like. Then beyond that it reinforced habits that I already have, but to continue with taking care of my sun protection. (Group 4)
	General conversations	I think right after the first test, I came back and briefly discussed it with my roommates. Beyond that, I haven't really had any conversations with anyone about skin care based on skin cancer. (Group 1)
	No communication	I haven't talked about this with anyone. (Group 1)
Cognitions/thoughts about tanning and/or sun protection behaviours	Change in frequency of thoughts about sun protection	I definitely find myself thinking about it a couple more times per week than usual … especially now with winter coming up, I think I'm thinking about it a lot more than I usually would during the wintertime, considering a lot of people don't think that you can get sun damage during the winter as you can also in the summer. (Group 2)
	Accountability with current skin protection behaviours	For the most part, it just reinforced to me to keep using SPF and that sort of thing. I care about it and I typically put sunscreen on when I'm going out in the sun in the summer and stuff, and I have SPF in my face lotion that I put on during the day. It just reinforced to make sure I use that lotion every day, and then thinking about sunscreen outside of just the summer. (Group 3)
	Recollection of basic information/application of knowledge	I don't know, mainly just the basic stuff like, ‘Oh, I should wear a sunscreen, or maybe I should and wear sunglasses or a hat, so I don't burn my scalp or something like that’ 'cause sometimes I forget about that, and then I burn my scalp outside. like a little debate like, ‘Oh, should I not? Do I really want to, kind of?’ if that makes sense. Just like, ‘I should do this, but I'm lazy, and I don't really want to. It's hot. Do I really wanna wear a long sleeve jacket or pants instead of shorts?’ Stuff like that. (Group 1)
	Contemplating behaviour change	…They always say, ‘Oh, when you get older, if you have a lot of sun, you can really tell on people’. ‘The more you take care of your skin when you're younger, the less age spots and other problems with your skin you have later’. That's pretty much what I talked about; every time that I was going outside, and it was sunny, I remembered the interview and the results. (Group 2)
	Future impact of skin cancer risk/prevention	…sounds really superficial, but what my skin would look like in the future and just how to keep it healthy because I wanna do everything I can to not contribute to the development of skin cancer in myself. More so just trying to keep my skin clear and healthy for the future for … when I'm older and my skin is not able to—I don't know—protect itself as much as it can now. (Group 2)
Individual risk perception	Underestimate of current risk	I learned that all of my freckles are sun damage. I don't know why I didn't make that connection. I guess, I didn't actually realize that people with fair skin were at a higher risk of skin cancer, and I have really fair skin. That was surprising, but it makes sense, logically. (Group 2)
	Confirmation of existing risk perception	I always thought that my risk was higher than most because of my skin and because of the color of my hair, and so I always had that kind of in the back of my head … but hearing that I was below average was nice to know that I have worked really hard for my—taking care of my skin, so it was relieving and validating. (Group 3)
	Change in risk perception as a result of the study leading to intention to change behaviour	I think I'm thinking about it a lot more than I usually would during the wintertime, considering a lot of people don't think that you can get sun damage during the winter as you can in the summer … I should continue to keep my body covered and put sunscreen on my face every morning, regardless if I'm going to be—knowing if I'm going to be in the sun or not. (Group 2)
	Impact of the sun on personal risk	I learned that I have a lot more sun damage than I thought. I had no idea that the sun had so much impact on my skin. I have naturally darker areas, and I was like, oh, it's probably just natural, but those were also the darkest areas on the picture. It shows that they had the most skin damage, which I thought was very interesting, that I can physically see how my skin has changed because of the sun. (Group 4)
	Change in mindset regarding skin cancer risk/prevention	I think my biggest philosophy has always just been, if I can get a base tan, I can prevent burning. I just feel like, for me, I just don't know—I feel like … I'm definitely more at risk, and I don't know if, after this study—I just feel more insecure about, man, I guess I should just be inside all the time. I don't know. (Group 1)
Actions taken by participants related to skin cancer prevention	Increased awareness of existing behaviour	Like I said, last week I definitely am thinking about taking protective measure a lot more. It was more of a wake‐up call, “Oh, I should stop treating it as lightly as I have, and treat it as a more serious subject.” I really try, you know, use sunscreen, use hats, and avoid the sun, learned a lot of the damages that I can't really have. (Group 2)
	Change in behaviour as a direct result of study participation	Once I learned the impact that it can have, I went out and bought sunscreen and bought some other things that I will need to protect my skin … I've been more aware of where I position myself. I often walk. If I have an option to walk in the shade when I'm outside, I do. (Group 2)
	Change in routine to accommodate skin protection	I did switch up my skincare routine a little bit, just trying to protect my skin from the sun, more so, on a daily basis because, historically, it's more like, if I was going out for the day, I would put on sunscreen and stuff like that. Now, I've been taking it by day even if I'm not gonna spend the whole day in the sun, but be mindful of taking care of my skin when I am outside even for shorter amounts of time. (Group 2)
	No action taken	I haven't done anything about it yet. I was going to take the charts and information that I got that was logged after my UV picture to my next dermatology appointment, which won't be for the next couple months, but just so that could be updated also in my files. (Group 2)
Thoughts/feedback on presentation of information through study	Overall satisfaction with material/study procedures	Oh, I was very satisfied with it. I learned a lot. Your study expounded on a lotta things that we inherently know but disregard as a society, so it was interesting to learn more about that information, and then also be someone that I can—I feel comfortable telling other people to take care of their skin … because of what I've learned. (Group 4)
	Desire for more specific information	It wasn't bad, by any means, but … it would've been really cool to understand more about skin cancer risk instead of just, do these things, 'cause it's like—I don't know. That's not for certain or anything. I'm not saying anything really is, but it would be cool to see exactly how sun exposure actually affects you or—you know what I mean? (Group 1)
	Feedback on surveys	The one thing that was a little bit longer than it might have needed to be were the surveys. I feel like a lot of them were worded—they were the same question asked over and over again, but just worded slightly different. (Group 2)
	Comments on randomization	I was kind of disappointed 'cause I was told there were three possible things I'd be able to participate in for this study … I think it was an actual test to see how likely you are for skin cancer. I actually would have been really interested in seeing [the picture] for myself 'cause I've had a history of damaging my face a bit … I would have liked to have seen the results that have currently affected me because of that. However, I just got the information, which I kind of already knew, so I was a little bit disappointed, but it's a study, so that makes sense. (Group 1)
	Comments on study procedures	I think it was very clear, and going to appointments and knowing what was needed of me was very clear, and the communication was very good. I didn't have any—I wouldn't have changed anything. (Group 2)
Suggestions for improvement	Tailor information to target audience	Maybe since the purpose of the study was to target college‐age students, you could have used more relatable images like, I don't know, from students at the university, if that makes sense. I don't know. (Group 1)
	Visual learning	I guess, me personally, I'm just a really visual thinker so having some sort of graphic that showed what the protections do. It would be cool if it was catered to each person. Like, “Oh, you have a severe case, this is almost what we recommend,” and having a graphic to show on that. That would have been cool. (Group 2)
	Desire for more in depth information	I think, for other participants, to keep the interest level relatively high as opposed to just simply going over a quiz about information. I feel like more personalized information or stuff about specific people's features involving skin cancer and skin care could have been really helpful. (Group 1)
	Interpretation of risk information	I actually wish that I had more information as to how to interpret the picture, because, I mean, I had a lot of little dots all over my face. I still had a lot of white patches as well. I tried to Google photographs of people that have bad skin versus good skin … I learned I have a lot of little sunspots and stuff. I don't know if that—my picture was good or bad or in between, to be completely honest. (Group 4)

## 3 RESULTS

In total, 38 students (mean age = 21.3 years, SD = 1.9; see Table 1 for demographic characteristics) participated. Approximately 42% (*n* = 16) participated in the fall and the remainder in the spring. Participants reported that different intervention components contributed to increased awareness of sun protection behaviours, shifts in cognitions about and motivation to implement sun protection strategies, and reported changes in the use of recommended skin cancer prevention strategies. The results are grouped under three sections: First, participants' experience with each intervention is described using the eight key categories and stratified by intervention. Next, the eight categories and subcategories (Table 3) are presented in Sections Objectives, 2 and Methods, 3, which cover internal reactions and external responses to the interventions and recommended changes to the interventions.

### 3.1 Experience with intervention components

#### 3.1.1 MC1R testing

The opportunity to be ‘tested for a genetic marker for skin cancer’ (Group 4) was appealing to participants who received *MC1R* testing, as many participants were unaware that variants could indicate an increased risk for developing skin cancer. However, participants reported a range of understandings of what variants in the *MC1R* gene represent. Interpretation of their risk results generally resulted in an underestimation of actual risk. For example, a response that reflected a more accurate understanding was, ‘I never really looked into, genetically, what my risk factors were, so that was all new. The results were that it was pretty unlikely that I am—I don't have the markers for it; however, I know that that's not a guarantee’ (Group 3). A response that reflected a more inaccurate understanding was, ‘It felt good because it assured me that I don't have any chance or maybe very little chance of having skin cancer. I felt good and assured that I'm not having skin cancer in the future’ (Group 3).

#### 3.1.2 UV photograph

Participants who received the UV photograph expressed emotional reactions—both positive and negative. Many participants reflected on a lack of previous understanding of the extent of sun damage that can be accrued even at a young age. Many participants viewed their individual UV photographs as a ‘wakeup call’, which led to increased awareness of current behaviours and motivation to improve their use of sun protection strategies. For example, ‘I learned that I have a lot more sun damage than I thought. I had no idea that the sun had so much impact on my skin. I have naturally darker areas, and I was like, oh, it's probably just natural, but those were also the darkest areas on the picture. It shows that they had the most skin damage, which I thought was very interesting, that I can physically see how my skin has changed because of the sun’ (Group 4). On the other hand, some expressed confusion regarding how to interpret the UV photograph including what is considered a ‘bad’ versus ‘good’ photograph, or what a UV image for someone their age should look like. One participant said, ‘I actually wish that I had more information as to how to interpret the picture, because, I mean, I had a lot of little dots all over my face. I still had a lot of white patches as well. I tried to Google photographs of people that have bad skin versus good skin … I don't know if that—my picture was good or bad or in between, to be completely honest’ (Group 4).

#### 3.1.3 Educational information

Participants in all groups described the general skin cancer prevention information as a useful reminder and confirmation of existing knowledge. ‘Oh, I was very satisfied with it. I learned a lot. Your study expounded on a lotta things that we inherently know but disregard as a society, so it was interesting to learn more about that information, and then also be someone that I can—I feel comfortable telling other people to take care of their skin … because of what I've learned’ (Group 4). Some described the information as too generic, common sense or repetitive and desired more in‐depth explanations rather than a ‘to‐do’ and ‘not‐to‐do’ list. ‘I actually probably would have liked to get more information from it. A lot of it was stuff I kind of already knew or that logically made sense. Just sun exposure would obviously give way to skin cancer’ (Group 1).

### 3.2 Internal reactions and external responses to the interventions

#### 3.2.1 Emotional reactions to personalized risk information

Participants reported a range of emotional reactions to receiving their personalized risk information (UV photo, *MC1R* results) but not the control education. Negative reactions included feelings of disappointment and guilt about current skin protective behaviours. ‘A little sad, a little disappointed in myself for not wearing sunscreen all the times I was supposed to’ (Group 2). Positive reactions included expressed relief about individual risk results, affirmation or validation of current skin protective behaviours, and increased confidence and self‐efficacy regarding skin cancer prevention. One participant noted ‘I was definitely relieved, but again, I know it's not a guarantee, and so it doesn't mean that I'm fine to just do whatever and not wear sunscreen. I recognize that’ (Group 3). Another participant expressed confidence in their ability to improve their skin cancer risk, ‘I felt that this is a problem, but it can be fixed…, yes, there's an issue here, but if we take proper precautions … then it will be okay’ (Group 2). Some participants reported expecting the risk results they received due to family history or expecting an opposite result (higher or lower risk) for the same reason.

#### 3.2.2 Existing knowledge or expectation of skin cancer risk versus novel information gained

Some participants described their experience with the intervention as a confirmation of existing knowledge or clarification of publicly accessible information. For example, ‘I felt like a lot of it was pretty basic information that I've learned in health classes as a kid. I think that it was done in a not frightening way. It was definitely more of just like a friendly reminder of things you should be doing, things you shouldn't be doing’ (Group 1). Others described alterations to their prior preconceptions about skin cancer risk and prevention, which they attributed to participation in the study, especially the *MC1R* test and the UV photographs. ‘I'm a frequent person that goes to the dermatologist and stuff like that, so I've always had my moles checked and my skin checked … I've never had a noticeably physical UV picture of my under skin noticed [never had a photograph taken that shows what my skin looks like underneath]’ (Group 2).

#### 3.2.3 Communication/information sharing

Participants most often cited family members, roommates and significant others as those with whom they shared the information gained from the interventions. For example, ‘I told my parents 'cause I thought it was interesting. Not that I was worried about it, but I have been keeping them kind of updated on the fact that I was in a research study about skin cancer and that I had that statistic [elevated above average *MC1R* result], and so I told my parents’ (Group 3). Reported information sharing between participants and others ranged from general discussions about skin cancer prevention to more specific sharing of personalized risk results. Participants who received a UV photograph or *MC1R* testing described having detailed discussions, such as sharing their personal risk results, having specific conversations about skin cancer risk, and educating others about the information gained in the study more than those who received education only.

#### 3.2.4 Motivation to act and actions taken

Some participants reported increased awareness and accountability of existing behaviours, including reports of ‘self‐checking’ how well they are using sun protection. One participant recalled a scenario when skin cancer prevention strategies should have been applied, but were not: ‘…I did think about it a lot on Spring Break, too. I was on a boat and I didn't have any sunscreen, didn't have anything on the surface of my skin. I was like, “Huh, this is not very good for me,” but I was like “Whatever” … I was like “Oh that's funny 'cause I was just barely doing a study about this. I'm not being very productive with it…”’ (Group 1). Participants' thoughts about sun protection behaviours ultimately reflected new motivation or lack of motivation. Participants who described feeling the increased motivation to change their behaviour attributed the change to concerns about ageing and the long‐term effects of the sun on their skin. One participant credited making the connection between current behaviour and impact on future skin cancer risk for increased motivation: ‘…sounds really superficial, but what my skin would look like in the future and just how to keep my skin clear and healthy for the future for, maybe, when I'm older and my skin is not able to—I don't know—protect itself as much as it can now’ (Group 2).

Many participants reflected on what they *should* change in their sun protection behaviours, rather than describing specific actions taken: ‘…It also just encouraged me to protect it better in the future to diminish my chance of getting [skin cancer]. I think I'll probably take those actions going forward’ (Group 2). Some participants reported specific changes in sun protection behaviours, such as changes in daily routines to accommodate skin protection, for example, ‘In the mornings I put on moisturizer … that has sunscreen in it. I made sure to do that. Then going outside, I've been a little bit more careful to bring my sunglasses, to try to wear a hat, those kinds of things. I think in general, overall I tend to be a little bit more cautious of the fact that skin cancer is a possibility. Whereas before, I don't think I really even took it into consideration that much’ (Group 3). Others described shifts in routines that they considered ‘off‐season’ that they attributed to the intervention, ‘I feel like … a lotta the things that I've done to prevent [skin cancer] go hand in hand with just the seasons changing, and so making sure that I'm covering up more, I think, just falls more in line with it's getting cold out, so of course, I'm gonna start doing those things. I guess they're easier to do when it's cold out if that makes sense. I haven't gone bed tanning though, so that's good’ (Group 1). While most reflected on cognitions and/or actions regarding skin cancer prevention, a few reported taking no action.

#### 3.2.5 Individual risk perception

Overall, participants reported having new and renewed understandings of their personal skin cancer risk. Participants described both accurate and inaccurate perceptions of their skin cancer risk. Some participants reported confirmation of their existing risk perception, due to their family history, outdoor activities, or skin tone. One participant reported an increase in their skin cancer risk perception, which led to an increased urgency to use sun protection. ‘…I thought I expected my skin to be damaged. I just came back from Africa … where I walked around all day in the sun where it was very, very hot. I never wore sunscreen, so I felt like, yes, that I would have some damage. I didn't know it was gonna be … that intense. It scared me enough to go out and purchase some things that will protect my skin’ (Group 2). One noted that while their overall risk perception did not change, they began to think differently about sun protection at different times of the year: ‘…especially now with winter coming up, I think I'm thinking about it a lot more than I usually would during the wintertime, considering a lot of people don't think that you can get sun damage during the winter as you can in the summer … I should continue to keep my body covered and put sunscreen on my face every morning, regardless if I'm going to be—knowing if I'm going to be in the sun or not’ (Group 2).

Many participants reported a shift in mindset regarding their skin cancer risk and prevention strategies. For instance, participants reported a new understanding of the impact of sun exposure on skin cancer risk and the level of sun damage that had already accumulated despite their young age. Those who had expressed a prior affinity for tanning attributed a shift in tanning philosophy to the intervention they received: ‘I think my biggest philosophy has always just been if I can get a base tan, I can prevent burning. I just feel like, for me … because of my weird situation, I'm definitely more at risk, and I don't know if, after this study—I just feel more insecure … I guess I should just be inside all the time. I don't know’ (Group 1).

### 3.3 Recommended changes to intervention content and delivery

#### 3.3.1 Targeting intervention content

Participants reflected on the importance of targeting intervention content to them, including information addressing college students' lifestyles, seasonal hobbies, and daily activities and using images in the intervention materials from college students. For example, one participant suggested, ‘Maybe a little bit more detail on the lifestyle stuff because even though I'm only slightly more at risk, I know that I'm out in the sun quite a bit with exercising and just working and stuff. I know that … increases my risk more than just my genetic material’ (Group 3). Another participant recommended including more skin cancer testimonials from college‐age individuals to increase engagement: ‘…especially if these are meant to help college students understand, almost personal testimonials of how skin cancer has affected someone, like if we got to watch a video or something about someone talking about their skin cancer story, 'cause I feel like all I really know about skin cancer is 50, 60‐year‐olds that I go to church with that are like, “Oh, yeah. This little Band‐Aid”—they have a wound or something. They're like, “Oh, yeah. I had to go get some skin cancer cut out.' It seems like very—I guess I just don't really know what having skin cancer is like”’ (Group 4).

#### 3.3.2 Visual learning

The concept of visual learning was mentioned in all interviews. Participants suggested providing more pictures or videos and using models that match the target demographic. ‘I think it would have been cool to have some more graphics that make it a bit more visual. I guess … I'm just a really visual thinker so having some sort of graphic that showed what the protections do. It would be cool if it was catered to each person. Like, “Oh, you have a severe case, this is what we recommend,' and having a graphic to show on that”’ (Group 2).

#### 3.3.3 Strategies to improve risk communication

As noted above, many participants who received the *MC1R* test or UV photograph reported wanting more information on how to interpret their risk, especially how their individual results compare to a ‘standard’ for their age group. For example, ‘…I didn't really know how to interpret it [the picture]. I wasn't satisfied not knowing really how damaged my skin was or if darker spots are worse than lighter spots. I wish I had more explanation about how to interpret the face scan’ (Group 4). Many participants desired more in‐depth educational information: ‘It would have been nice to get just something a little more in‐depth because all the things I was told, I already knew … just from day‐to‐day life and parents telling me to put on sunscreen in the sun. It would have been nice if maybe it was quantified…’ (Group 2).

## 4 DISCUSSION

This qualitative study explored undergraduate student perspectives on receipt of skin cancer preventive interventions delivering personalized risk information. We found that different forms of risk communication could not only increase reported awareness of sun protection behaviours, but could also instigate communication about risk results and skin cancer prevention with others, and contribute to reported shifts in motivation to implement sun protection strategies. In some cases, the risk communication interventions contributed to students' reported changes in skin cancer prevention and risk behaviours. While all participants expressed that the general skin cancer prevention information was a useful reminder, those who received a UV photograph or *MC1R* genetic test results demonstrated higher engagement with their risk results, which included more detailed discussions with others and specific intentions or actions taken to change skin cancer prevention behaviours. Costs for these technologies have decreased immensely over the years. Genetic testing, for example, is now offered direct‐to‐consumer. Similarly, smartphone apps and other technologies to increase the portability of and access to UV photography are currently being developed,^52^, ^53^ which will increase the feasibility of administering these interventions.

Emotional and behavioural reactions to the intervention were often a reflection of participants' prior understanding of their individual risk (e.g., family skin cancer history and biological risk factors). However, participants reported that receiving a UV photograph, in particular, provided a much higher ‘shock factor’ than the *MC1R* test results did. Participants who received a UV photograph tended to report stronger intentionality toward specific changes in their sun protection habits. While these findings are consistent with previous UV photograph studies that focus on appearance and photo‐ageing effects of sun exposure,^21^, ^22^, ^23^, ^24^ to our knowledge, a connection between UV photographs and perceived skin cancer risk has not been described. Participants reported a more positive experience when they received personalized risk information. However, it was notable that for some, their interpretations of the *MC1R* genetic test results and the UV photographs led to the underestimation of their overall skin cancer risk. These same participants expressed a desire for more guidance on how to use the risk information they received.

The timing of intervention delivery could be worth exploring further, as skin cancer risk and preventive behaviours could differ by season. For example, some participants in the fall expressed a lack of urgency to engage in sun protection as the weather was getting colder. They also reported being less likely to think about sun protection in the winter. On the other hand, participants in the spring reported increased awareness of individual sun exposure and sun protection strategies as they were getting closer to summer. Participants from the spring provided more detailed responses than those from the fall, especially on topics such as individual risk perception and cognitions about skin cancer and sun protection behaviour. This could be due to the approaching summer season, which put sun exposure at the forefront of students' minds.

### 4.1 Limitations

A few limitations of the current study should be noted. First, the majority of participants in this sample were non‐Hispanic, White females from a single geographic location and university, which limits the generalizability of the results. In addition to diversifying geographic location and race/ethnicity in samples, future work could examine the association between qualitative responses and participants' reported behavioural outcomes in a mixed methods study. Another potential limitation is that participants who were willing to complete the interview might have been more engaged, which could inflate conclusions about satisfaction with the interventions. While we did not find a significant trend in one direction or another among participants, it is possible that negative emotionality from personalized results or confusion about the results may have contributed to participants' willingness to complete the interview as well.

### 4.2 Future directions

Our findings suggest several potential directions for future skin cancer prevention interventions—both the content and the delivery—in the college student setting. Targeting materials to this demographic will likely increase engagement and satisfaction with the intervention. Specifically, the models and images used in educational materials should match the target audience. We found that the most common recommendations for improving intervention content and delivery focused on utilizing more graphics and images to appeal to ‘visual learners’ as well as testimonials, pictures and videos of the skin cancer experience within the young adult demographic. Based on our findings that students reported satisfaction with, interest in, and engagement with the information provided through all three of the intervention components, it appears that the use of personalized risk information within the context of a skin cancer prevention intervention for college students holds promise and could be tested in larger studies. A recent qualitative study by Khan et al.,^54^ noted that the way in which genetic risk information is delivered—whether in numeric form or in lay terms—can affect comprehension of personal genetic risk results, which was reflected in the experience of our study participants. Some participants could benefit from follow‐up with a genetic counsel and/or dermatologist to discuss and clarify misinterpretations in risk information results as needed. Based on the current findings, the UV photograph and *MC1R* risk information appear to deliver distinct but complementary personally relevant information. These skin cancer prevention interventions could be affected by seasonal differences and those seasonal effects should be accounted for within the context of interventions or in study designs. The findings also suggest that potential intervention mechanisms or mediators investigate in future studies include perceived risk for skin cancer and emotional reactions to intervention content.

### 4.3 Conclusions

In conclusion, we conducted a qualitative study that examined undergraduate students' overall experience with participating in an intervention that provided skin cancer education as well as personalized skin cancer risk information in the form of a UV photograph and/or *MC1R* genetic testing. Our findings indicate that students are responsive to risk‐related multicomponent interventions and they show some motivation and intentionality toward changing skin cancer risk behaviour. Additional work to modify the interventions based on participant feedback, examine how qualitative perceptions relate to intervention outcomes of interest (e.g., sun protection behaviour change), and address potential seasonal influences on intervention outcomes is needed to confirm and expand on these findings.

## AUTHOR CONTRIBUTIONS

All authors made substantial contributions to the conception or design of the work, or the acquisition, analysis, or interpretation of data for the work; assisted in drafting the work or revising it critically for important intellectual content; gave final approval of the version to be published; and are in agreement to be accountable for all aspects of the work in ensuring that questions related to the accuracy or integrity of any part of the work are appropriately investigated and resolved.

## CONFLICT OF INTEREST

The authors declare no conflict of interest.

## ETHICS STATEMENT

Our study protocol was approved by the human research ethics boards at the University of Utah.

## Data Availability

The data that support the findings of this study are available from the corresponding author upon reasonable request.
